# Temporal and spatial genetic differentiation in the crab *Liocarcinus depurator* across the Atlantic-Mediterranean transition

**DOI:** 10.1038/srep29892

**Published:** 2016-07-19

**Authors:** Marta Pascual, Ferran Palero, Víctor Hugo García-Merchán, Enrique Macpherson, Aymée Robainas-Barcia, Francesc Mestres, Tania Roda, Pere Abelló

**Affiliations:** 1 Dept. Genètica, Microbiologia i Estadística and IRBio, University of Barcelona, Av. Diagonal 643, 08028 Barcelona, Spain; 2 Centre d’Estudis Avançats de Blanes (CEAB-CSIC), Carrer d’Accés a la Cala Sant Francesc 14, 17300 Blanes, Spain; 3 University of Quindio (Group of Evolution, Ecology and Conservation, EECO), Armenia, Quindío (Colombia).; 4 Institut de Ciències del Mar (ICM-CSIC), Passeig Marítim de la Barceloneta 37-49, 08003 Barcelona, Catalonia, Spain

## Abstract

Spatial genetic studies often require sampling broadly separated areas, difficult to access simultaneously. Although comparing localities surveyed at different time periods might result in spurious genetic differentiation, there is a general believe on the stability of genetic structure through time, particularly if sampled localities are isolated or very distant. By analysing spatial and temporal genetic differentiation of the portunid crab *Liocarcinus depurator* we assessed the contribution of historical and contemporary processes on population connectivity patterns across three main oceanographic discontinuities along the Atlantic-Mediterranean transition: Gibraltar Strait, Almeria-Oran Front and Ibiza Channel. A partial fragment of the *cytochrome oxidase I* gene was sequenced in 366 individuals collected from localities at both sides of each discontinuity during three time periods. Although localities showed genetic fluctuations through time, a significant gradient was detected along the coast for all sampling periods. Significant inter-annual differences identified within the Alicante area, north of the Almeria-Oran Front, were associated with shifts in the relative contribution of Atlantic and Mediterranean water masses. The persistence of a clinal pattern in the Atlantic-Mediterranean transition area together with local fluctuations suggests a complex balance of dispersal and selection.

Spatial genetic studies have increased in the last decades in many taxa while temporal genetic studies, despite being essential to understand biodiversity distribution, are still scarce. Landscape genetics and phylogeography are both concerned with understanding the distribution of genetic variation across natural environments^1^,^2^. Landscape genetic papers mostly focus on the effects of barriers or environmental variables reducing connectivity through contemporary gene flow^3^,^4^. On the other hand, phylogeographic studies often ascribe genetically differentiated units to old vicariant events^5^,^6^ or to adaptation (reviewed in Knowles^7^). Both spatial genetic approaches require sampling different areas, sometimes broadly separated, which are difficult to survey simultaneously. Indeed, most studies include samples collected over different years whereas others do not even mention collection dates^8^,^9^. It has been claimed that different genetic markers are appropriate for drawing inferences at different temporal scales, with mitochondrial DNA, chloroplastic DNA or nuclear gene sequences being most suitable to unveil historical processes and microsatellite or amplified fragment length polymorphism loci for contemporary processes^10^. However, the importance of the sampling scheme should not be forgotten regardless of the marker used. The relative importance of historical and current processes on the spatial distribution of genetic diversity is difficult to ascertain, since both time and space may not be independent. Consequently, studies sampling the same areas during multiple time periods should be carried out to identify whether the observed genetic variation is due to historical and/or contemporary processes, and demonstrate whether mitochondrial DNA markers are suitable or not to describe present day connectivity patterns.

The large population sizes of marine organisms generally result in little genetic differentiation despite restricted gene flow, and molecular markers show practical limitations in making inferences for recent timescales and single time point samples^3^. Dispersal is expected to be mostly related to pelagic larvae being driven by oceanographic mesoscale processes because many marine species present homing behaviour and restricted movement in the adult phase at mid/large geographical scales^11^,^12^. However, multi-species comparisons across the same environmental gradient failed to find a significant relationship between population genetic differentiation and planktonic larval duration^13^,^14^,^15^. The observed discrepancies between theory and data could be caused by the scarce knowledge on behaviour patterns and/or different evolutionary processes (e.g. vicariance, colonization or selection events) affecting the studied species^16^, but they could also be due to the sampling scheme used if there were inter-annual fluctuations of water masses across oceanographic fronts.

Three oceanographic discontinuities along the Atlantic-Mediterranean transition in the south and eastern Iberian Peninsula (Gibraltar Strait, Almeria-Oran Front and Ibiza Channel) have been shown to restrict gene flow in several marine species. The connection of the Mediterranean Sea and the Atlantic Ocean through the Gibraltar Strait is characterized by the inflow of surface Atlantic water and outflow of deeper Mediterranean water^17^,^18^. Significant genetic differences between populations located at both sides of Gibraltar Strait have been observed in some crustacean and fish species^9^,^14^,^19^,^20^. The Almeria-Oran front is a large scale density front located 400 km east of Gibraltar Strait and formed by the convergence of the main jet of incoming Atlantic water and the Mediterranean Sea. It is controlled by the geographic position and strength of the Eastern Alboran Gyre^21^ and it has been proposed as the main genetic break between the Atlantic Ocean and the Mediterranean Sea^16^. At the Ibiza Channel, northwestern Mediterranean water recirculates northwards following the Balearic coast so that just a small fraction of the Northern Current actually crosses the channel^22^. This front is thought to significantly reduce gene flow between populations, but disparate effects are found depending on the species^12^,^14^,^23^,^24^. The dynamic behaviour of these fronts, with significant intra- and inter-annual variability^18^,^25^,^26^, can affect gene flow across these discontinuities and influence population differentiation levels^12^,^27^.

Previous studies in the portunid crab *Liocarcinus depurator* showed that the Almeria-Oran front did not represent a significant barrier to gene flow, whereas Ibiza Channel acted as the main genetic barrier^14^. Such unexpected result was attributed to historical and/or contemporary processes. In order to characterize the stability of marine genetic barriers through time we have explored spatial and temporal differentiation in *L. depurator* across the three main oceanographic discontinuities along the Atlantic-Mediterranean transition. A partial region of the mitochondrial DNA *cytochrome oxidase subunit I* (*COI*) was analysed in 366 individuals from five areas situated at both sides of each discontinuity (Gibraltar Strait, Almeria-Oran front and Ibiza Channel) and sampled during three time periods (Fig. 1). We found that diversity decreases from Atlantic to Mediterranean populations and identified steep changes in allele frequency in the transition between the two basins. Most interestingly, genetic diversity was found to vary across years, which indicates that both historical and contemporary processes affecting population genetic structure can be unveiled using mitochondrial DNA markers when temporal samples are used.

## Results

Genetic diversity levels varied across space and time (Table 1). Haplotype richness was significantly different among localities (ANOVA test: F = 9.03, P = 0.003). Diversity was higher in the two populations under Atlantic water influence (CADI and WALB) and decayed in the Mediterranean (ALAC and VALE), with the lowest values found in DELT (Table 1). Isolation by distance patterns were observed for the 2013 (Mantel test: r = 0.94, P = 0.006) and for the 2009–2010 (Mantel test: r = 0.80, P = 0.027) datasets, whereas no significant pattern was obtained for the 2005–2007 samples (Mantel test: r = 0.63, P = 0.112). Nevertheless, García-Merchán *et al*.^14^ reported significant isolation by distance in those 2005–2007 samples when including an additional locality (north from the area selected in the present work), which suggests that our non-significant pattern could be related to the lower number of comparisons included in the present work.

Genetic differentiation among years was significant in CADI and ALAC (Fig. 2). Differences in ALAC were driven by the 2007 sample, while samples from the other two years did not differ genetically (Table 2). For CADI, significant differentiation was observed between the samples from 2010 and 2013. The first axis of the principal coordinate analysis plot, constructed using pairwise F_ST_ values between all populations and sampling years, explained 88% of the observed variance and split the samples into two main groups (Fig. 3), one group including all samples from populations with Atlantic influence (CADI and WALB) plus the 2007 sample of ALAC, and another group including all remaining samples.

For all three oceanographic discontinuities, most comparisons between adjacent localities were significant (Table 3). Nevertheless, these effects were not permanent, since at least one comparison across every oceanographic discontinuity produced no genetic differentiation during the three sampling periods. Finally, no genetic differentiation was observed between the localities which are not separated by any known oceanographic front (i.e. VALE and DELT).

The haplotype network (Fig. 4) resulted in two main star shape groups each centrally including a highly frequent haplotype (Ldep03 and Ldep02). These two frequent haplotypes had been previously shown to describe opposite geographic frequency clines^14^. Along the new time series dataset analysed here, most CADI individuals also belonged to the first group (Fig. 4 and Supplementary material), while most individuals from DELT belonged to the second group. Similarly, the Bayesian phylogenetic tree obtained with all *Liocarcinus depurator* haplotypes recovered two highly supported clusters, one including mostly haplotypes from areas with a strong Atlantic influence (Ldep03 and related haplotypes), and another including haplotypes mostly found in the Mediterranean (Ldep02 and related haplotypes) and were thus referred as the Atlantic (ATL) and the Mediterranean (MED) haplogroups, respectively (Fig. 4 and Supplementary material). The mean frequency of the two haplogroups was estimated for each year and locality, and frequency distributions were obtained by resampling 1000 times (with replacement) the two haplogroups within each population taking into account sample size. Each haplogroup maintained its frequency relatively constant through time in some localities but not in others (Fig. 5), in agreement with the global Kst results per locality (Fig. 2). For instance, the mean (±s.d.) frequency of the MED haplogroup showed little variation between years both in DELT (90 ± 3%) and in WALB (37 ± 2%). However, for the other three localities (CADI, ALAC and VALE) the frequency of the two haplogroups changed through time (Fig. 5), as indicated by the larger standard deviation of the mean between the three sampling years (ranging from 12 to 26%). The relative frequency of the two groups changed the most within ALAC, which presented 55% of individuals with the ATL haplogroup in the 2007 sample while the proportion was less than 14% in the other two sampling years. The higher frequency of haplotypes belonging to the ATL haplogroup (Fig. 5) would explain why ALAC07 was more similar to the localities most influenced by Atlantic waters in the principal coordinate analysis (Fig. 3).

When analysing the presence/absence of Atlantic or Mediterranean waters in the different sampling areas and years (Table 1), we observed the presence of typical Atlantic waters (salinity ca. 36.5‰) in the area of the Gulf of Cádiz (CADI) and Alboran Sea (WALB), in agreement with other studies^18^,^26^. The areas north of Ibiza Channel, Gulf of Valencia (VALE) and Ebro Delta (DELT) were dominated as expected by Mediterranean waters (>37‰). However, an increase of Atlantic waters was observed north of Almeria-Oran front (ALAC area) during the winter 2006, whereas this area was dominated by Mediterranean waters in winter 2008 and 2012. The entrance of Atlantic waters through the Almeria-Oran front in winter 2006 suggests that *Liocarcinus depurator* larvae could have drifted with that current, indicating that the higher frequency of adults with ATL haplogroups observed in 2007 could result from contemporary connectivity mediated by temporal changes in water currents.

## Discussion

In contrast to the single-frame fixed picture available until now, our temporal sampling reveals that mitochondrial DNA phylogeographic patterns can vary significantly across time and space in marine species. All three oceanographic discontinuities restrict larval dispersal between populations, but the lack of genetic differentiation during particular time periods indicates that the effect of oceanographic barriers might be eventually relaxed. The significant interannual genetic differentiation and haplotype diversity observed in some localities further indicate that both historical and contemporary processes can affect population genetics in marine species, with temporal mesoscale events explaining observed variations in connectivity patterns. Despite diversity and population structure of a species are largely shaped by historical events (i.e. glacial periods) the present study evidences that contemporary processes impact local genetic diversity and modify connectivity between populations. Temporal variability of oceanographic discontinuities should not be overlooked when defining connectivity patterns because this could mislead conservation decisions (e.g. when designing networks of marine protected areas)^28^,^29^.

Changes in environmental conditions (estimated from paleoclimatic data) and historical shifts of species distributions can shape genetic variation, as shown by spatially-explicit demographic modelling of genetic differentiation^30^. The higher genetic diversity levels observed in *Liocarcinus depurator* populations under strong influence from Atlantic waters and decaying diversity going into the Mediterranean, suggests an Atlantic origin of the species. Many phylogeographic studies identify genetically differentiated units that can be attributed to vicariance or selection reducing gene flow^31^. Nevertheless, divergence patterns may also carry the signature of altered contemporary landscapes together with historical ones^32^. Coalescence times of *COI* haplotypes for *L. depurator* can be related to an abrupt decline of sea temperature in north Atlantic waters around 38–40 kya^14^,^33^. Therefore, the two main haplogroups observed in *L. depurator* probably diverged due to vicariance events and posterior range expansions, as observed in other species influenced by climatic fluctuations during the Quaternary^34^,^35^,^36^.

**Figure 1:**
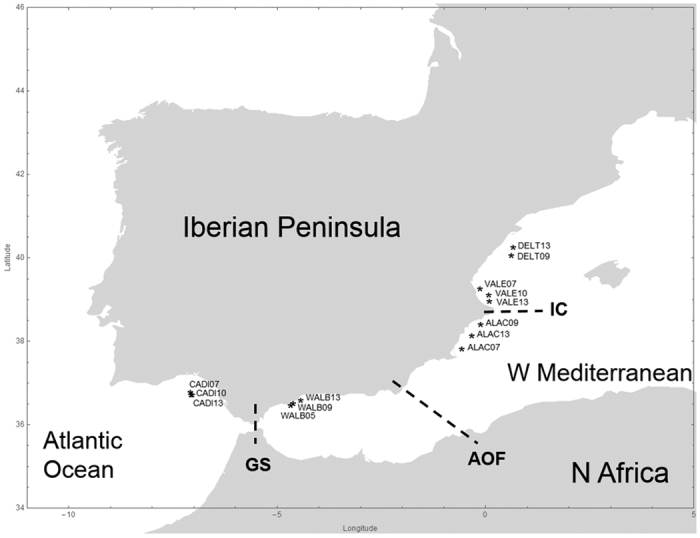
Sampling locations of *Liocarcinus depurator* along east and south of the Iberian Peninsula. Each location was sampled several years. Each mark represents the mean latitude and longitude of all the individuals analysed from a campaign. Location acronyms are DELT (Delta de l’Ebre), VALE (València), ALAC (Alacant), WALB (Málaga) CADI (Cádiz) and the two last digits of the sampling year. Dashed lines identify the main oceanographic discontinuities present in the area: Gibraltar Strait (GS), Almeria-Oran Front (AOF) and Ibiza Channel (IC). Figure created using the software Mathematica 10.4.

**Figure 2:**
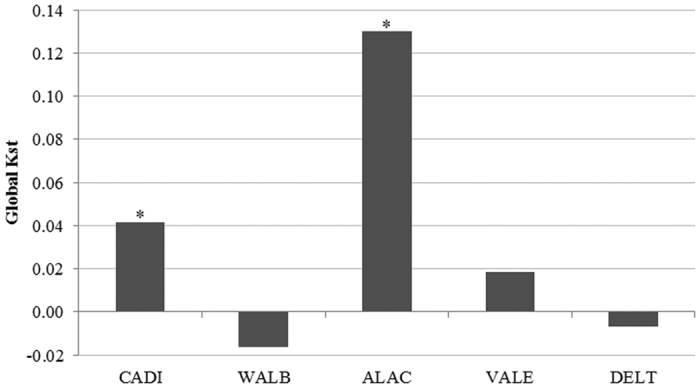
Global genetic differentiation (Kst) among years for each locality (*P < 0.05). Acronyms as in Fig. 1.

**Figure 3:**
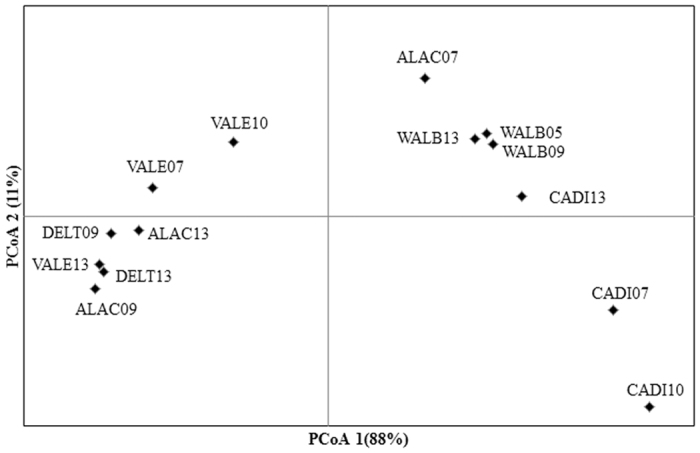
Principal Coordinates Analysis obtained from the genetic distance matrix of F_ST_ values between all years and localities. The two digits identify the sampling year. Acronyms as in Fig. 1.

**Figure 4:**
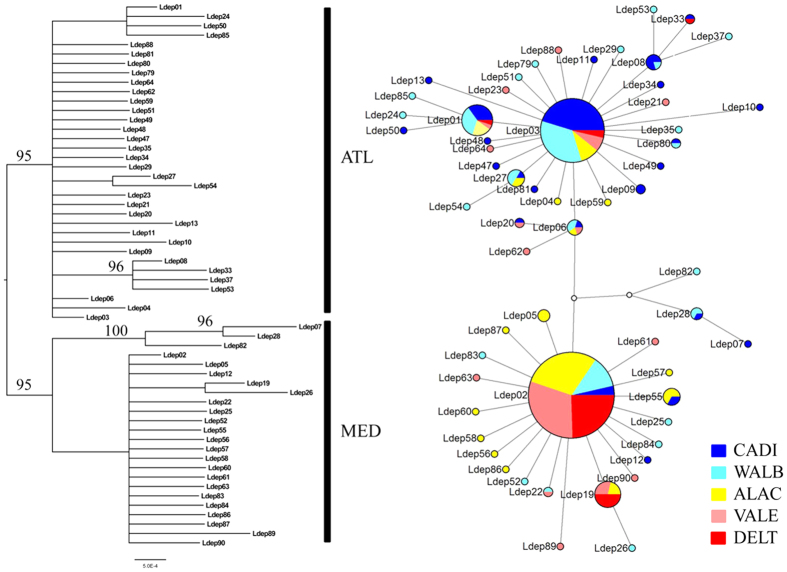
Bayesian haplotype tree for the *COI* sequences of *Liocarcinus depurator* (Ldep). Only posterior probability values higher than 95% are given. The median joining haplotype network combines the frequency of haplotypes from different years for each locality. Each circle represents a haplotype and its size is proportional to its frequency. Populations are colour coded as indicated in the legend and their acronyms are as in Fig. 1.

**Figure 5:**
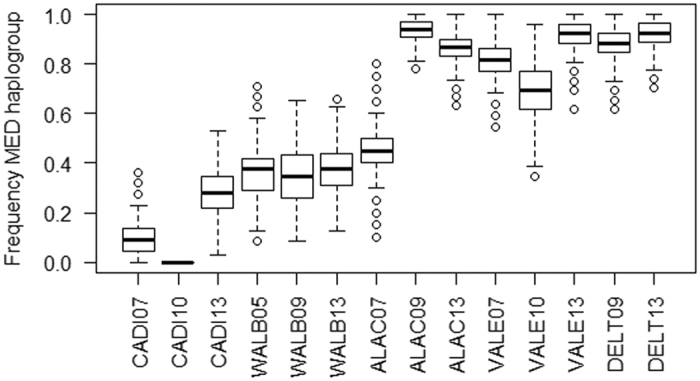
Proportion of individuals assigned to the MED haplogroup in each location and sampled year from 1000 pseudoreplicates. Acronyms as in Fig. 1.

The greater diversity observed in samples under stronger Atlantic influence (Gulf of Cádiz, CADI and Alboran Sea, WALB, Fig. 1) might as well correspond to higher effective population sizes or admixture between genetically differentiated groups. In fact, WALB samples were the most diverse both in terms of haplotype and nucleotide diversity and presented the two haplogroups at intermediate mean (±s.d.) frequencies across years (37 ± 2% of the MED haplogroup). This observation suggests that, even if historical processes may explain the genetic distances observed among haplotypes, the relative frequency of those haplotypes in the population is mostly affected by contemporary processes and connectivity patterns. For example, the epipelagic water transport into the Mediterranean at the Gibraltar Strait is larger and less variable across time than the deeper outflow towards the Atlantic Ocean^37^, so that *Liocarcinus depurator* epipelagic larvae could drive genetic connectivity of this species by moving along with surface currents^38^. This seems to be in accordance with previous studies relating ocean circulation and directional gene flow across discontinuities^4^,^12^. Similarly, the higher interannual genetic variation of CADI is also in agreement with the higher standard deviation of outward waters through Gibraltar Strait reported by Astraldi *et al*.^37^.

The largest fluctuations in haplotype frequency across time were found in ALAC located between two main oceanographic discontinuities, the Almeria-Oran front in the south and the Ibiza Channel in the north. The ALAC07 sample showed a relatively high frequency of individuals from the Atlantic (ATL) haplogroup, whereas ALAC09 and ALAC13 were clearly dominated by haplotypes from the Mediterranean (MED) haplogroup. A strong genetic break between Almeria-Oran front and Ibiza Channel and higher proportion of Atlantic genotypes was also observed in the comber (*Serranus cabrilla*) using microsatellite loci suggesting genetic exchange from the west side of the Almeria-Oran front towards the east and north^12^. Nonetheless, two of our samples showed a low frequency of individuals belonging to the ATL haplogroup, which indicates that this exchange is not stable through time. Several processes could explain the observed interannual genetic differences in the ALAC area. Given that our samples are somehow disperse over space we cannot discard the existence of micro-spatial genetic structuring due to the coastal boundary layer influencing larval dispersal^39^. The interannual variation in ALAC could be also mediated by the oceanographic variability observed along the Ibiza Channel. According to the oceanographic model by Balbín *et al*.^40^ the Northern Current could flow through the Ibiza channel in mild winters while effectively blocking the channel during cold winters. Nevertheless, no correlation was found here between the years where the Ibiza Channel was blocked^25^ and the presence of the ATL haplogroup in the populations located at both sides of Ibiza Channel (VALE and ALAC). Therefore, the higher presence of ATL haplotypes in ALAC07 seems to be most probably related to the incoming Atlantic water through the Almeria-Oran front during the previous winter as indicated by the match in salinity levels.

Temporal variations in genetic differentiation patterns in *COI* have also been found in the sea urchin *Paracentrotus lividus*^27^, with a marked change in 2008 caused by an arrival of many private haplotypes coincident with an abnormality in the circulation pattern around the Ibiza Channel. Temporal genetic variation has been assessed using nuclear markers in a few marine organisms suggesting a complex interplay of selection and drift. For instance, significant differences were detected among years in the blue crab *Callinectes sapidus* in the northern Gulf of Mexico, but changes were observed mostly at one locality and attributed to larval mortality after an oil spill^41^. Similarly, significant differentiation was observed between discrete size classes of the Caribbean spiny lobster *Panulirus argus* that could be related to changes in dispersal through currents or selective processes affecting larval survival^42^. Temporal genetic shifts in the bicolour damselfish (*Stegastes partitus*) pointed to the effect of variable reproductive success, and highlighted the unpredictable nature of connectivity in coral reef fishes^43^,^44^. Oceanographic connectivity, as well as temperature and salinity differences between sites, contribute to genetic differentiation in the Atlantic herring *Clupea arengus*^45^. Thus, reduced connectivity could result from restricted gene flow through oceanographic discontinuities but also from selection. The different impact of oceanographic discontinuities as barriers to gene flow in species with similar population sizes and dispersal capabilities made Bierne *et al*.^46^ to suggest that they represent tension zones (i.e. endogenous barriers to gene flow) trapped by the discontinuity. Many marine species may have had their Mediterranean and Atlantic populations separated during glacial periods, with genetic differentiation maintained after secondary contact. Following Bierne *et al*.^46^, genetic differentiation would be maintained in those tension zones only because a genetic barrier is superimposed on the natural barrier. This interesting hypothesis would explain the presence of two groups in *Liocarcinus depurator* as well as in other species with high dispersal capabilities (e.g. *Palinurus elephas*^3^), but remains to be tested using a multiple-marker approach.

In conclusion, our study evidences that not only historical but also contemporary processes determine population genetics patterns in marine organisms. Some localities present larger interannual variations than others, probably due to changes in ocean circulation shifts that modify gene flow across fronts. All three oceanographic discontinuities analysed here varied in their intensity through time and should be considered permeable. Thus, discrepancies on the genetic-barrier effect of oceanographic discontinuities in different species could result from differences in the sampling periods. Further studies with longer time series analyses and multiple markers should be carried out to better characterize the changes in gene flow taking place through space and time.

## Materials and Methods

*Liocarcinus depurator* is a temperate-cold portunid crab commonly found on the continental shelf and upper slope of the north-east Atlantic and Mediterranean Sea, with a peak of reproduction during winter in the Mediterranean and summer in colder latitudes of the northern Atlantic Ocean^47^,^48^. Females reach maturity during their first year of life and 2–3 year age classes can be observed in wild populations^47^. To avoid using individuals from different cohorts within the same year, the analysed samples were selected by mean (±s.d.) carapace size (41 ± 6 mm) which would correspond to one year old mature individuals. Therefore, the main dispersal phase (planktonic larvae) originating our sampled individuals would have occurred during the previous winter every year.

### Study area and sample collection

Samples were obtained from the MEDITS_ES^49^ and ARSA^50^,^51^ fishery research surveys by bottom trawl gear. Several hauls were carried out for each of five areas chosen in order to analyse the effect of the three oceanographic discontinuities mentioned above. Sampling collections, coded by their acronym and year of sampling, and oceanographic discontinuities are shown in Fig. 1, made using the CountryData and Graphics functions in Mathematica v10.4 software^52^. Cádiz (CADI) is located west of the Strait of Gibraltar, Málaga (WALB) between the Strait of Gibraltar and the Almeria–Oran Front, Alacant (ALAC) between the Almeria–Oran Front and the Ibiza Channel, and València (VALE) and Delta de l’Ebre (DELT) are both located north of the Ibiza Channel. To assess the interannual variability on the genetic composition of the populations, samples from three years were analysed for each locality, with the exception of DELT where only two sampling years were available (Table 1). Data for 2005 and 2007 were used in García-Merchán *et al*.^14^ and data for years 2009, 2010 and 2013 are original contributions for the present study. The number of individuals analysed for each area and year range between 20 and 32 individuals (Table 1).

#### DNA extraction, amplification and sequencing

Muscle tissue from each individual was preserved in pure ethanol on board of the vessel. Total DNA extraction was performed for 2009 and 2010 samples using Chelex 10% as in Estoup *et al*.^53^, and samples from 2013 were extracted with QIAamp® DNA Mini Kit (Qiagen Inc) following the manufacturer’s instructions. The *cytochrome oxidase I* (*COI*) gene fragment was amplified using the universal primers LCO1490 and HCO2198^54^. PCR amplifications and sequencing for 2009 and 2010 samples were carried out as described in García-Merchán *et al*.^14^. PCR reactions for 2013 samples were carried out in 20 μl final volume with 1 μl of genomic DNA, 1 U of Taq polymerase (GoTaq, Promega), 4 μl 5 × buffer (GoTaq, Promega), 1 μl MgCl_2_ (25 mM), 0.5 μl dNTP’s (1 mM) and 0.4 μl (10 μM) of each primer. PCR products of 2013 samples were cleaned with ExoSAP (1.2 U Exonuclease and 1.2 U Shrimp Phosphatase per μl) at a proportion of 1:2 (ExoSAP:PCR product) and sequenced at the Scientific and Technical Services of the University of Barcelona (Spain) or at Macrogen Inc. (Seoul, Korea).

#### Data analyses

Sequences were visually inspected, aligned and trimmed, to obtain a final alignment of 527 bp, with BioEdit v7.2.5^55^. The number of haplotypes, haplotype diversity and nucleotide diversity and their standard deviations were calculated using DnaSP v5^56^. In order to allow for comparison among samples with different number of individuals, haplotype richness was computed with rarefaction methods as implemented in the software Contrib 1.02^57^. To evaluate differences in haplotype richness between localities, an ANOVA test was performed after ensuring normality of the data using STATISTICA v8.0. Global genetic differentiation among sampling years for the same locality were estimated measuring the Kst statistic^58^ and its significance obtained through a permutation test with 1000 replicates as implemented in DnaSP. Pairwise genetic differentiation among sampling areas and sampling years were estimated as pairwise F_ST_ values and their significance assessed performing 1000 permutations with Arlequin v. 3.5^59^. *P*-values were corrected for multiple comparisons using the false discovery rate (FDR) as in Narum^60^. To visualize the relationship among different samples in a two-dimensional space a Principle Coordinate Analyses (PCoA) was performed based on F_ST_ pairwise distances with GenAlEx v6.5^61^. The same software was used to analyse isolation by distance in each sampling period comparing pairwise genetic and geographical distances with a Mantel test. The geographical distances were measured along the 200 m isobath as in García-Merchán *et al*.^14^.

The phylogenetic relationships between *Liocarcinus depurator* haplotypes were estimated by Bayesian Inference (BI) using MrBayes v 3.2^62^. We sampled across the entire GTR model space with invgamma using two independent runs of 1 million generations each. Markov chains were sampled every 100 generations and a default burn-in value of 25% was used after ensuring convergence of the two chains. The haplotype network for *L. depurator COI* sequences was reconstructed using the median joining method^63^ as implemented in NETWORK V 5 (fluxus-engineering.com).

#### Oceanographic data

Sea surface salinity provides presence/absence data of Atlantic and Mediterranean water masses along the different areas and years in the western Mediterranean^18^,^25^,^26^. In the present study we obtained from SOCIB^64^ (Balearic Islands coastal ocean observatory and forecasting system, http://www.socib.eu/) and buoys located within the different sampling areas (Spanish Harbours “Puertos del Estado”; www.puertos.es) salinity data as a proxy for identifying Atlantic and Mediterranean water masses (Table 1). We averaged salinity during the winter period of the previous year, which corresponds to the peak of reproduction and larval abundance of the adults sampled the following year.

## Additional Information

**Accession codes**: *Liocarcinus depurator* haplotypes are available in GenBank under accession numbers JN564801-JN564829 and KU941953- KU941986.

**How to cite this article**: Pascual, M. *et al*. Temporal and spatial genetic differentiation in the crab *Liocarcinus depurator* across the Atlantic-Mediterranean transition. *Sci. Rep.* **6**, 29892; doi: 10.1038/srep29892 (2016).

## Supplementary Material

Supplementary Information

## Figures and Tables

**Table 1:** Sampling locality, year of collection, number of analysed individuals (N), number of different haplotypes (Nh) and genetic diversity measures of each sample of *Liocarcinus depurator*.

Population	Year	N	Nh	Haplotype richness	Haplotype diversity	Nucleotide diversity	Salinity (PSU)
DELT	2013	27	4	2.48	0.430	0.0017	38.1
	2009	26	4	2.68	0.348	0.0016	38.0
VALE	2013	26	6	3.85	0.354	0.0016	38.0
	2010	26	9	6.61	0.732	0.0035	38.1
	2007	22	6	4.55	0.411	0.0021	37.5
ALAC	2013	30	9	6.02	0.600	0.0026	37.8
	2009	32	9	5.24	0.488	0.0016	37.5
	2007	20	6	5.00	0.789	0.0038	36.5
WALB	2013	32	12	7.82	0.817	0.0043	37.0
	2009	23	10	8.09	0.846	0.0046	36.5
	2005	24	9	6.83	0.746	0.0040	36.3
CADI	2013	32	13	8.81	0.877	0.0045	36.5
	2010	24	8	5.83	0.507	0.0014	36.5
	2007	22	10	8.27	0.710	0.0029	36.2

**Table 2:** Genetic differentiation across time. Pairwise F_ST_ and P values between samples collected from the same locality in different years.

Locality	Pairwise comparison	Fst	(P value)
DELT	2009–2013	−0.014	0.566
	2007–2010	0.002	0.293
VALE	2007–2013	−0.001	0.410
	2010–2013	0.066	0.078
	2007–2009	**0.329**	0.000
ALAC	2007–2013	**0.212**	0.006
	2009–2013	−0.005	0.403
	2005–2009	−0.028	0.882
WALB	2005–2013	−0.027	0.959
	2009–2013	−0.019	0.736
	2007–2010	0.004	0.315
CADI	2007–2013	0.031	0.090
	2010–2013	**0.109**	0.003

**Table 3:** Effect of the oceanographic discontinuities on genetic differentiation across space and time in the Atlantic-Mediterranean transition.

Oceanographic discontinuities	Samples compared	Fst	(P value)
Gibraltar Strait	CADI07–WALB05	0.074	0.032
	CADI10–WALB09	**0.173**	0.000
	CADI13–WALB13	−0.005	0.458
	WALB05–ALAC07	−0.016	0.496
Almería-Oran Fron	WALB09–ALAC09	**0.407**	0.000
	WALB13–ALAC13	**0.278**	0.000
	ALAC07–VALE07	**0.181**	0.004
Ibiza Channel	ALAC09- VALE10	**0.085**	0.017
	ALAC13–VALE13	−0.010	0.504
No discontinuity	VALE10–DELT09	0.030	0.167
	VALE13–DELT13	−0.003	0.437
