# Supplementary material for: Temporal and spatial genetic differentiation in the crab Liocarcinus depurator across the Atlantic-Mediterranean transition
Source: Sci Rep. 2016 Jul 19;6:29892. doi: 10.1038/srep29892 (PMC4949458; doi:10.1038/srep29892)
Supplement: Supplementary Information [file srep29892-s1.pdf]

Supplementary Material

Temporal and spatial genetic differentiation in the crab *Liocarcinus depurator* across the Atlantic-Mediterranean transition

Marta Pascual, Ferran Palero, Víctor Hugo García-Merchán, Enrique Macpherson, Aymée Robainas-Barcia, Francesc Mestres, Tania Roda and Pere Abelló

Supplementary material. Haplotype frequencies in each locality and sampling year. Haplotype sequences are available at GenBank under the accession numbers JN564801-JN564829 and KU941953- KU941986.

Haplotype	CADI07	CADI10	CADI13	WALB05	WALB09	WALB13	ALAC07	ALAC09	ALAC13	VALE07	VALE10	VALE13	DELT09	DELT13
Ldep01	2	1	4	1	3	3	3		1		1		1	
Ldep02			6	6	7	7	7	23	19	17	13	21	21	20
Ldep03	12	17	9	11	6	12	6	1	1	1	4	1	2	1
Ldep04							1							
Ldep05							2		1					
Ldep06	1					2	1				1			
Ldep07	1													
Ldep08	1		3		1									
Ldep09	1	1												
Ldep10	1													
Ldep11	1													
Ldep12	1													
Ldep13	1													
Ldep19								1	2		3	1	2	5
Ldep20			1							1				
Ldep21										1				
Ldep22						1				1				
Ldep23										1				
Ldep24				1										
Ldep25				1										
Ldep26				1										
Ldep27			1	1	1	1			2					
Ldep28			1	1		1								

Haplotype	CADI07	CADI10	CADI13	WALB05	WALB09	WALB13	ALAC07	ALAC09	ALAC13	VALE07	VALE10	VALE13	DELT09	DELT13
Ldep29				1										
Ldep33			1											1
Ldep34			1											
Ldep35					1									
Ldep37		1												
Ldep47		1												
Ldep48		1												
Ldep49		1												
Ldep50		1												
Ldep51					1									
Ldep52					1									
Ldep53					1									
Ldep54					1									
Ldep55			2					2	2					
Ldep56								1						
Ldep57								1						
Ldep58								1						
Ldep59								1						
Ldep60								1						
Ldep61											1			
Ldep62											1			
Ldep63											1			
Ldep64											1			
Ldep79			1											
Ldep80			1			1								
Ldep81			1											
Ldep82						1								
Ldep83						1								
Ldep84						1								

Haplotype	CADI07	CADI10	CADI13	WALB05	WALB09	WALB13	ALAC07	ALAC09	ALAC13	VALE07	VALE10	VALE13	DELT09	DELT13
Ldep85						1								
Ldep86									1					
Ldep87									1					
Ldep88												1		
Ldep89												1		
Ldep90												1		
N	22	24	32	24	23	32	20	32	30	22	26	26	26	27
